# Flavonoids as Antioxidants and Developmental Regulators: Relative Significance in Plants and Humans

**DOI:** 10.3390/ijms14023540

**Published:** 2013-02-07

**Authors:** Cecilia Brunetti, Martina Di Ferdinando, Alessio Fini, Susanna Pollastri, Massimiliano Tattini

**Affiliations:** 1 DiSPAA, Department of Agri-Food and Environmental Science, University of Florence, Viale delle Idee 30, 50019 Sesto Fiorentino (FI), Italy; E-Mails: cecilia.brunetti@unifi.it (C.B.); martina.diferdinando@unifi.it (M.D.F.); alessio.fini@unifi.it (A.F.); 2 IPP, Institute for Plant Protection, National Research Council, Via Madonna del Piano 10, 50019 Sesto Fiorentino (FI), Italy; E-Mail: pollastri@ipp.cnr.it

**Keywords:** auxin movement, chloroplast flavonoids, dihydroxy B-ring-substituted flavonoids, MAPK, nuclear flavonoids, reactive oxygen species (ROS), signaling molecules

## Abstract

Phenylpropanoids, particularly flavonoids have been recently suggested as playing primary antioxidant functions in the responses of plants to a wide range of abiotic stresses. Furthermore, flavonoids are effective endogenous regulators of auxin movement, thus behaving as developmental regulators. Flavonoids are capable of controlling the development of individual organs and the whole-plant; and, hence, to contribute to stress-induced morphogenic responses of plants. The significance of flavonoids as scavengers of reactive oxygen species (ROS) in humans has been recently questioned, based on the observation that the flavonoid concentration in plasma and most tissues is too low to effectively reduce ROS. Instead, flavonoids may play key roles as signaling molecules in mammals, through their ability to interact with a wide range of protein kinases, including mitogen-activated protein kinases (MAPK), that supersede key steps of cell growth and differentiation. Here we discuss about the relative significance of flavonoids as reducing agents and signaling molecules in plants and humans. We show that structural features conferring ROS-scavenger ability to flavonoids are also required to effectively control developmental processes in eukaryotic cells.

## 1. Introduction

Flavonoids, the vast class of secondary metabolites encompassing more than 10,000 structures, have long been reported as serving multiple functions in plants suffering from a wide array of environmental injuries [1–7]. Flavonoids have indeed the capacity to absorb the most energetic solar wavelengths (*i.e.*, UV-B and UV-A), inhibit the generation of reactive oxygen species (ROS) and then quench ROS once they are formed [8].

**Figure 1:**
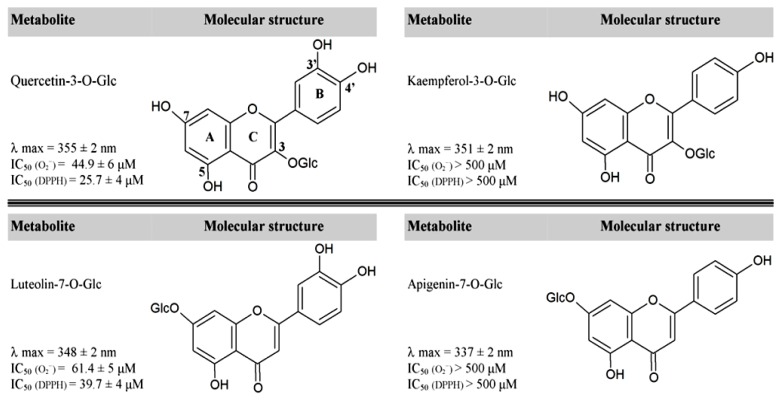
Absorbance maxima and ROS-quenching capacity of mono-(apigenin and kaempferol) and dihydroxy B-ring-substituted (quercetin and luteolin) flavonoid glucosides (glc). Absorbance spectra were recorded in phosphate buffer at a metabolite concentration of 50 μM. IC_50_ denotes the molar concentration required to reduce by 50% the concentrations of superoxide anion (O_2_^−^) and the synthetic free radical DPPH (2,2-diphenyl-1-picrylhydrazyl) as estimated following the protocols reported in [10,17].

However, recent evidence suggests that UVB-induced flavonoid biosynthesis is not for primarily fulfilling UV-screening functions [6,8]. Flavonoids indeed replace hydroxycinnamic acid derivatives—which have greater absorbing capacities than flavonoids over the 280–320 nm spectral region—in cells exposed to high sunlight irradiance [8–10]. Actually, light-responsive flavonoids are the dihydroxy B-ring-substituted forms, such as quercetin 3-*O* and luteolin 7-*O*-glycosides, and not the monohydroxy B-ring-substituted counterparts, such as apigenin 7-*O* and kaempferol 3-*O*-glycosides (Figure 1) [10–13]. Dihydroxy B-ring-substituted flavonoids have a greater antioxidant capacity, but not a greater ability to absorb UV-wavelengths than their monohydroxy B-ring-substituted counterparts (Figure 1). Recent findings of dihydroxy B-ring-substituted flavonoids being located within or in the proximity of ROS generation centers lead to hypothesize for flavonoids a fundamental antioxidant role in photoprotection [8,14,15]. This hypothesis conforms to flavonoid biosynthesis being up-regulated by a plethora of abiotic and biotic stresses [8,16,17], which have in common the generation of ROS [18].

Flavonoids have long been also suggested to perform reducing functions in humans. There is vast literature showing that in many food species the activities of enzymes involved in the flavonoid biosynthesis as well as their antioxidant potential increase greatly as a consequence of changes in environmental conditions [19–24]. Nevertheless, the significance of this antioxidant activity *in vivo* has been recently questioned [25–28]. In humans, flavonoids undergo intracellular metabolism, e.g., conjugation with glutathione, and circulating flavonoids are usually *O*-methylated or glucuronidated [25]. These structural modifications decrease the ability of flavonoids to donate hydrogen atoms [29]. We also note that flavonoids are poorly soluble in the aqueous cellular milieu (e.g., flavonoids in plant cells are usually glycosylated to enhance their solubility in the aqueous cellular milieu), and the concentration of flavonoid in the plasma or most tissues range from high nanomolar (nM) to low micromolar (μM). This poses, once time more, the still unsolved question of the potential ROS-scavenging properties of flavonoids as determined in *in vitro* experiments (in which aglycones are usually tested), with their actual significance *in vivo*, both in humans and in plants [8,30–36]. Nevertheless, mild stress conditions may significantly increase the concentration of bioactive flavonoid forms in different foods, e.g., quercetin derivatives [19–24]. This issue is far from being conclusively addressed and will require further research aimed at identifying and quantifying individual flavonoids at inter- and intra-cellular levels.

In recent years, flavonoids have been reported as serving signaling functions in eukaryotic cells, through their ability to interact with a range of protein kinases that supersede key steps of cell growth and differentiation [28,33–35]. These functional roles of flavonoids may be of great value in plant photoprotection [1,6,8,36], and a strong correlation between flavonoids and the plant hormone auxin has been conclusively proven [37,38]. Interestingly, the health beneficial effects of flavonoids in humans are also thought to reside mostly on their ability to control the activity of several protein kinases, including the mitogen activated protein kinases (MAPK) [31].

In this brief review article, we discuss about the relative significance of flavonoids as antioxidants and developmental regulators in plants and humans. We show that flavonoids may preserve humans from several diseases and exert a developmental control in plants through very similar action modes.

## 2. ROS-Scavenging Functions in Plants and Humans: Similarities and Differences

The concentration and the inter- and intra-cellular locations of flavonoids in leaves exposed to high-light stress are suitable for antioxidant functions [8]. As stated above, in response to excess light stress—in the presence or in the absence of UV-radiation—the biosynthesis of antioxidant flavonoids is almost exclusively enhanced [17,39–41]. It is a part of the folklore of plant photobiology that the ratios of quercetin to kaempferol derivatives or luteolin to apigenin derivatives steeply increase in response to UV-B, UV-B + UV-A, or PAR (photosynthetic active radiation, over the 400–700 nm waveband) irradiance [11,12]. Antioxidant flavonoids in healthy leaf cells indeed must have a dihydroxy B-ring-substitution [10]. The most reactive OH-groups, for example the 7-OH in flavones or the 3-OH-group in flavonols, are indeed glycosylated (Figure 1). Glycosylation increases solubility in the aqueous cellular milieu, preserves the reactive OH-groups from auto-oxidation [5,42], allows the transport of flavonoids from the endoplasmic reticulum to various cellular compartments, and their secretion to the plasma membrane and the cell wall [43–46]. As a consequence, the hydrogen-donating ability of the flavonoid forms usually encountered in healthy plant cells resides almost exclusively on the presence of catechol group in the B-ring of the flavonoid skeleton [8,10].

**Figure 2:**
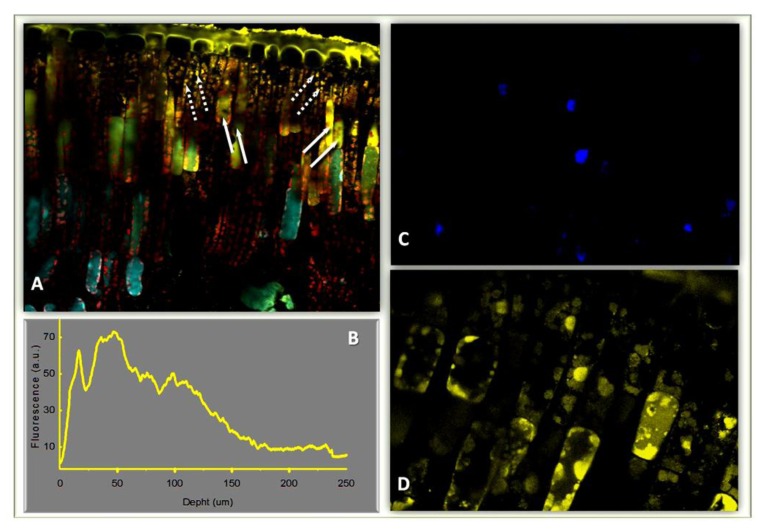
Inter and intracellular distribution of dihydroxy-B-ring-substituted flavonoid glycosides in leaves of *L. vulgare* leaves acclimated to full sunlight irradiance. Cross sections were stained with Naturstoff reagent and fluorescence images recorded in a Confocal Laser Scanning Microscope flowing the protocols of [10]. In brief, specimens were excited at 488 nm and fluorescence recorded at 580 ± 10 nm: this excitation-emission set-up has been previously reported to exclusively visualize flavonoids, in particular flavonoids with a catechol group in the B-ring of the flavonoid skeleton [10]. Dihydroxy flavonoids accumulate mostly in the adaxial tissue (up to a distance of 150–170 μm from adaxial surface, as revealed by profiles of their fluorescence acquired at 580 nm, reported in (**B**). Dotted and solid white arrows in (**A**) indicate flavonoids in the chloroplast and the vacuole, respectively. Dihydroxy flavonoids are located in the nucleus of mesophyll cells. Nuclei were stained with both DAPI (to visualize the nucleus) and Naturstoff reagent and images were recorded in the blue-channel for detecting DAPI fluorescence (**C**) or the yellow-channel for detecting flavonoid fluorescence (**D**).

Recent evidence shows that antioxidant flavonoids are located in the nucleus of mesophyll cells, and hence capable of quenching H_2_O_2_ and H_2_O_2_-generated hydroxyl radical (Figure 2C,D) [8,20,24,47,48]. Dihydroxy B-ring-substituted flavonoid glycosides have a great capacity to complex Fe and Cu ions, which catalyzes the formation of hydroxy radical in the presence of H_2_O_2_, through the well-known Fenton reaction (Fe(II)/Cu(II)+H_2_O_2_ → Fe(III) + HO^−^ + HO**^•^**). Antioxidant flavonoids are also located within centers of ROS generation, *i.e.*, the chloroplast (Figure 2A) [8,14,49], and they have been shown to quench singlet oxygen *in vivo* [14]. These flavonoids are apparently associated with the chloroplast outer envelope membrane (OEM) and may limit the diffusion of H_2_O_2_ and singlet oxygen out of the chloroplast, following the excess excitation energy-induced depletion of other ROS-scavengers (SOD, APX, carotenoids, isoprene, and tocopherols) [8,50–54]. It is worth noting that flavonoids may preserve the chloroplast OEM from oxidative damage. Flavonoids are indeed capable of stabilizing membranes that contain non-bilayer lipids, such as monogalactosyl diacyl glycerol (MGDG), and this may be important for the cytoplasmic side of OEM that is poor in MGDG [55,56]. Quercetin 3-*O*-rutinoside [57] may interact with the polar head of phospholipids at water–lipid interface, thus enhancing membrane rigidity [58], and consequently preserving membranes from oxidative damage [59].

Flavonoids in the vacuole of mesophyll cells (Figure 2A) are in very high concentrations [60] and hence capable of removing H_2_O_2_ freely diffusing out of the chloroplast (or the peroxisomes) under severe excess light stress, when the activity of APX (or CAT) is strongly depressed [51,54,61]. Flavonoid glycosides have a much smaller affinity than corresponding aglycones for peroxidases, but their concentrations may allow detoxify H_2_O_2_ efficiently [8,17,60]. We therefore suggest that the reducing functions of flavonoids are of primary significance in plants suffering from severe stress conditions. These functional roles are consistent with the very high concentration of dihydroxy B-ring-substituted flavonoids usually detected in plants adapted to high sunlight irradiance [10,62–64]. Based on previous experiments [60], we estimate that the concentration of flavonoids may be in the range 50–100 μM, on a whole-leaf level. Since 80% of antioxidant flavonoids are usually located in adaxial both epidermal and palisade parenchyma cells (Figure 2A,B), their vacuolar concentration may be as high as 100–200 μM in these cells [60]. Based on recent data of Ferreres *et al.* [60] we calculate that 200 μM quercetin 3-*O*-rutinoside may scavenge as much as 45 μM H_2_O_2_ s^−1^. We also note that because of the small volumes of the nucleus and the chloroplast envelope membrane, the molar concentrations of antioxidant flavonoids in these compartments may be high enough to detoxify ROS efficiently.

The significance of flavonoids as antioxidants in humans requires much more caution. For example, Ishige *et al.* [65] have shown that flavonoids may protect neuronal cells from oxidative damage through mechanisms other than ROS quenching. Quercetin diminishes oxidative damage by enhancing the concentration of glutathione [65]. In addition, quercetin blocks Ca^2+^-influx: this allows cells to survive at low levels of cytoplasmic Ca^2+^, thus blocking Ca^2+^-channels responsible for cell death. Multiple mechanisms of action have been also recently proposed in mediating cardiovascular effects of flavonoids. These include metal complexation, inhibition of xantine oxidase activity (which generates superoxide anions) as well as chemical quenching of ROS [32].

We note that the antioxidant potentials of flavonoids in animal cells have been tested in *in vitro* experiments. Since an antioxidant has been defined as a molecule that diminishes oxidative stress at low concentrations [66], it is difficult to discriminate among the mechanisms and the mode of actions through which the oxidative stress is actually countered. As a consequence, further research is to be performed to conclusively address the actual significance of flavonoids as ROS quenchers in the highly integrated network of antioxidant defenses operating in humans [26,27,31]. For example, flavonoids have been reported to break free radical chain reactions in lipids (by donating hydrogen atoms to lipids or lipid peroxyl radicals), with quercetin having similar reduction potential of ascorbate and much lower than α-tocopherol [31]. Rutin has significantly smaller capacity than quercetin, but greater ability than kaempferol aglycone to reduce lipid radicals. These findings corroborate previous suggestions on the crucial role of the catechol group in the B-ring of the flavonoid skeleton in conferring free radical quenching capacity to flavonoids [25]. Furthermore, the reaction rate of quercetin (and rutin) with peroxyl radical in plasma is approximately one order magnitude greater than those of ascorbate and α-tocopherol [31]. Nevertheless, the concentration of quercetin in plasma may be as low as just 0.6% and 1% of ascorbate and tocopherol, respectively [67–69]. As a consequence, the free radical scavenging or more in general the ROS quenching hypothesis for flavonoids in plasma and most other tissues is severely constrained.

It is not surprising that plants being sessile organisms have developed much more efficient mechanisms to quench ROS as compared with animals. Plants may suffer from massive generation of ROS not only on a daily, but also on a seasonal basis, as a consequence of an excess of radiant energy reaching the photosynthetic apparatus [70]. Oxidative stress due to an excess of excitation energy in the chloroplast may be exacerbated under conditions that limit the diffusion of CO_2_ to the carboxylation sites as well as the efficiency for CO_2_ carboxylation [7,8,15]. These environmental constraints to CO_2_ assimilation rate include drought/salinity, low/high temperature, and nutrient scarcity. These are the very conditions that may greatly reduce the activity of enzymes primarily aimed at detoxifying ROS in the chloroplast [50,51,61,71,72], while strongly up-regulating the biosynthesis of ROS-scavenging flavonoids. Flavonoids have been suggested as constituting a secondary antioxidant system in plant tissues exposed to vastly different stressors [8,16,73].

## 3. Flavonoids Play Key Functions as Developmental Regulators-Signaling Molecules in Plants and Humans

Flavonoids in low concentrations are capable of displaying functional roles of extraordinary significance in plant-environment interactions [6,74]. We note that during the colonization of land by plants the metabolism of dihydroxy B-ring-substituted flavonoids was already active. The whole set of genes for the biosynthesis of luteolin and quercetin derivatives has been detected in liverworts and mosses [75]. These findings lead to the hypothesis that it is unlikely that flavonoids fulfilled primary UV-B screening functions when early plants moved from water to colonize the land [1,3,6]. As already mentioned, dihydroxy B-ring-substituted flavonoids have a greater capacity than most other flavonoids to scavenge ROS, and do not display a superior capacity to absorb the shortest solar wavelengths. Actually, early land plants suffered from water and nutrient shortage in an O_2_-rich atmosphere under high-light conditions [76]. As a consequence flavonoid, particularly flavonol biosynthesis protected plants from the concomitant action of different environmental constraints, being high-UV-B just one of these.

To fully accomplish ROS-quenching activities flavonoids have to be in the high μM range. It has been authoritatively suggested [1,77] that flavonoid concentrations in early terrestrial plants were probably too low for serving efficiently ROS-quenching and UV-B screening activity (for which millimolar (mM) concentrations are necessary) [8]. Flavonoids in the nM range may regulate auxin movement and catabolism [38]. The ability of flavonoids to establish auxin gradients translates into phenotypes with strikingly different morpho-anatomical traits [37,72,78]. Flavonoids at the plasma membrane are effective inhibitors of PIN and MDR-glycoproteins that are involved in the cell-to-cell movement of auxin (see below for details). In addition, flavonoids have regulatory functions on the activity of IAA-oxidase, with strikingly different effects depending on their chemical structure [79,80]. Relatively recent evidence of a nuclear location (Figure 2) of flavonoids (as well as of enzymes of flavonoid biosynthesis), is consistent with flavonoids being capable of regulating the activity of proteins responsible for cell growth [75,81]: flavonoids may therefore act as transcriptional regulators [82].

A short-PIN protein—PIN5—has been recently discovered in the endoplasmic reticulum, the site of flavonoid biosynthesis [83,84]. This finding, when coupled with the observation that PIN5 is the only PIN detected in early terrestrial plants [83] opens new scenarios on the functional roles of flavonoids not only in early but also in current-day terrestrial plants [8]. It is conceivable that flavonoids served, and still likely serve primary functions as developmental regulators in plants experiencing a wide range of environmental injuries. To test this hypothesis an in-depth analysis of biological activity, knowledge of the chemical structure relationships of flavonoids is urgently needed. The ability of flavonoids to inhibit the activity of the efflux facilitator PIN and MDR proteins (see below) depends on the presence of the catechol group in the B-ring of the flavonoid skeleton. Indeed quercetin is a much more potent inhibitor than kaempferol of the polar auxin transport (PAT) [30,85].

The control of flavonoids on auxin movement may have great value in the stress-induced morphogenic responses (SIMR) of plants, the flight strategy of sessile organisms exposed to unfavorable environments [86–88]. Species rich in dihydroxy flavonoids display phenotypes with strikingly different morphological features as compared with those rich in monohydroxy flavonoids [81,89]. Dwarf-bushy phenotypes are usually encountered in sunny environments, and offer few, small and thick leaves to direct sunlight irradiance, thus protecting leaves located deep in the canopy from light-induced severe perturbation of cellular homeostasis. In contrast shaded plants, which are rich in kaempferol and/or apigenin derivatives (while displaying negligible concentrations of quercetin derivatives [10,13,17,63] display long internodes, great leaf lamina size coupled with reduced leaf thickness [86]. We also note that flavonols may profoundly alter the root architecture and serve important functions in plant/arbuscular mycorrhizal fungi symbiosis for a review see [90]. This interaction between two eukariotes represented a key innovation that helped plants adapt to the terrestrial environment during their initial colonization of land, as water and nutrient shortages were likely the first challenges plants faced on land [76,91,92].

Actually, SIMR may contribute to diminishing the oxidative damage driven by excess radiant energy at organ and whole-plant levels (an antioxidant function, *sensu* Halliwell) [26]. Thus, chemical features conferring ROS-quenching capacity are also responsible for the ability of flavonoids to regulate the development of individual organs and the whole plant. In plants these functional roles are not in conflict, because require dramatically different concentrations and inter/intracellular locations.

In humans, flavonoids behave as developmental regulators/signaling molecules as well [25,27,33,93]. As stated above the cellular effects of flavonoids in eukaryotic cells may be mediated by their interactions with specific proteins central to intracellular signaling cascades (for a review see Hou and Kamamoto) [28]. Flavonoids may inhibit signaling pathways involved in cell growth and differentiation by directly binding to the ATP catalytic sites of protein kinases [28]. Quercetin forms hydrogen bonds with Ser212 through the 3′-OH group, thus showing a greater capacity than kaempferol to inhibit mitogen-activatived protein kinase kinase (MEK1) activity [94]. Interestingly, one of the most selective inhibitors of lipid kinase signaling cascades, such as phosphoinositide 3-kinase (PI3K) cascade, has been modeled on the structure of quercetin [95]. Protein kinases (mostly MAPKs) have now been suggested to control the expression of antioxidant enzymes, inhibit cell cycle progression and cell proliferation, and the expression and functional activation of oncogenes [96–98]. Therefore protein kinases have been now suggested as molecular targets for chemoprevention by flavonoids [99], and flavonoids exert their chemopreventive effects acting as regulators of oxidative stress-induced protein kinase signaling pathways, more than as conventional hydrogen-donating antioxidants. This brief presentation is not aimed at discussing in depth the structural features enabling flavonoids to modulate the activities of ATP-binding sites, activation loops or allosteric sites of protein kinases [28]. However, we note that most experiments have analyzed aglycones in modeling interactions between flavonoids and ATP-binding sites of a wide range of protein kinases. This still poses the question why the most active OH-groups in the flavonoid skeleton are being silenced in eukaryotic cells through different metabolic pathways [25,99]. As long reported the hydroxylation pattern in the B-ring of the flavonoid skeleton, *i.e.*, the 3′,4′-hydroxylation, is crucial in conferring regulatory properties to flavonoids upon the activities of a wide range of proteins [25,28]. Furthermore, chemical features responsible for hydrogen-donating capacities also confer the ability to flavonoids to regulate key steps in cell growth and differentiation in eukaryotic cells.

The flavonoid-MAPK relationships in plants are an intriguing, still unexplored issue that may link the action modes of flavonoids in humans and plants. The MAPKs are activated in response to free radical-induced injury in cells and contribute to the processes of cellular survival or death [31]. In plants, MAPK cascades are involved in auxin response, and serine/threonine protein kinases mediate light-dependent differential growth response in plants [34]. The propagation of auxin signals in the aerial parts of the plants is indeed through a PINOID serine-threonine protein kinase (PID). PID belongs to the plant specific AGCVIII kinases and regulates the asymmetrical subcellular localization of PINFORMED (PIN) auxin efflux facilitator protein that is responsible for the establishment of auxin gradients and maxima [100]. At the plasma membrane, PID partially co-localizes with PIN. PIN and Multidrug Resistant (MDR)/P-glycoproteins exhibit a tight control on cell-to-cell movement of auxin by acting individually or perhaps in concert [101–103]. Flavonoids have the capacity to inhibit the activity of PID, PIN and MDR-glycoproteins (in a very similar fashion to MDR-proteins in mammals, [104]). Plasma membrane as well as cell wall-located flavonoids may regulate the efflux of auxin by interfering with plasma membrane PIN/MDR glycoproteins, and a wide array of wall associated kinases (WAK). WAKs allow cells to recognize and respond to their extracellular environments, and profoundly alter the cell shape [105]. MAPK signaling cascades may constitute a second messenger in receptor-like kinases-induced control of growth and differentiation of cells in different plant organs [106]. This is consistent with MAPKs being involved in the transduction of oxidative signals (e.g., H_2_O_2_ accumulation) [107], and in repressing the auxin responsive promoters [108].

A raf-like MAPKKK gene confers drought tolerance in rice by enhancing the activity of peroxidase: it is possible that flavonoids are involved in this response, as flavonoids are preferential substrates for peroxidases [109]. MAPKs are capable of activating antioxidant enzymes in plants, e.g., MKK1/MPK6 induce up-regulation of catalase in *Arabidopsis* [110]. Protein kinases display very similar functions in humans [97]. These findings suggest that the MAPKs are not only activated by ROS but may also regulate ROS levels in eukaryotic cells [111,112].

Noticeably, flavonoid biosynthesis likely follows oxidative stress events, as MYB transcription factors that regulate the biosynthesis of flavonoids are activated by changes in redox homeostasis. Recently, ABA has been shown to regulate the biosynthesis of flavonoids, and MAPK cascade functions in ABA signaling, and to be downstream of ABA-induced ROS production [113,114]. Noteworthy, ABA may serve similar functions in plants and animals [115], and this plant hormone may have served functional roles, during the evolution of early land plants, that go beyond the mere ability to regulate stomatal opening and seed germination, and being upstream of flavonoid biosynthesis [116,117].

## 4. Conclusions

Flavonoids serve a multiplicity of functions in eukariotic cells not only because of their location in different cells and sub-cellular compartments, but also as a consequence of their chemical structures. Stress-responsive flavonoids display a great potential to reduce various forms of reactive oxygen species, a common condition to which plants are faced with when experiencing different stresses of abiotic and biotic origin. Antioxidant flavonoids may contribute greatly to ROS-detoxification through chemical ROS quenching in plant cells, whereas in human cells, their functions as reducing agents appears of relatively minor significance. However, flavonoids may serve similar functions in plants and humans (in high nanomolar to low micromolar range) by tightly regulating the activities of different protein kinases, which, in turn, are responsible for mediating ROS-induced signaling cascades vital to cell growth and differentiation. These functional roles are effectively served by ROS-quenching flavonoids. The flavonoid-protein kinases relationships, which have been extensively investigated in humans, have not yet received attention in plants. Here we note that MAPKs are activated by oxidative stress signals and mediate responses of plants to vastly different stressors. Flavonoids have the potential to greatly affect MPAK signaling cascades in plants not only by directly binding to the active sites of the proteins, but also modulating their activation through ROS-scavenging activities.
